# Four years after the implementation of antimicrobial stewardship program in Jordan: evaluation of program’s core elements

**DOI:** 10.3389/fpubh.2023.1078596

**Published:** 2023-05-30

**Authors:** Samar Khaled Hassan, Eman Zmaily Dahmash, Thaira Madi, Omar Tarawneh, Tuqa Jomhawi, Worood Alkhob, Rola Ghanem, Zina Halasa

**Affiliations:** ^1^Department of Accreditation, Healthcare Accreditation Council, Amman, Jordan; ^2^Department of Chemical and Pharmaceutical Sciences, School of Life Sciences, Pharmacy and Chemistry, Kingston University London, Kingston upon Thames, United Kingdom; ^3^Department of Consultation, Healthcare Accreditation Council, Amman, Jordan; ^4^Laboratory Directorate, Ministry of Health, Amman, Jordan; ^5^Clinical Pharmacy Directorate, Ministry of Health, Amman, Jordan

**Keywords:** antimicrobial resistance, antimicrobial stewardship program, WHO, low- and middle-income countries (LMICs), Jordan

## Abstract

**Objectives:**

To combat antimicrobial resistance, the World Health Organization (WHO) urged healthcare organizations in Low- and Middle-Income Countries (LMICs) to implement the core elements of the antimicrobial stewardship (AMS) programs. In response, Jordan took action and developed a national antimicrobial resistance action plan (NAP) in 2017 and commenced the AMS program in all healthcare facilities. It is paramount to evaluate the efforts to implement the AMS programs and understand the challenges of implementing a sustainable and effective program, in Low-Middle Income Country (LMIC) contexts. Therefore, the aim of this study was to appraise the compliance of public hospitals in Jordan to the WHO core elements of effective AMS programs after 4 years of commencement.

**Methods:**

A cross-sectional study in public hospitals in Jordan, using the WHO AMS program core elements for LMICs was carried out. The questionnaire comprised 30 questions that covered the program’s six core elements: leadership commitment, accountability and responsibility, AMS actions, education and training, monitoring, and evaluation, and reporting and feedback. A five-point Likert scale was employed for each question.

**Results:**

A total of 27 public hospitals participated, with a response rate of 84.4%. Adherence to core elements ranged from (53%) in the leadership commitment domain to (72%) for AMS procedure application (actions). Based on the mean score, there was no significant difference between hospitals according to location, size, and specialty. The most neglected core elements that emerged as top priority areas were the provision of financial support, collaboration, access, as well as monitoring and evaluation.

**Conclusion:**

The current results revealed significant shortcomings in the AMS program in public hospitals despite 4 years of implementation and policy support. Most of the core elements of the AMS program were below average, which requires hospital leadership commitment, and multifaceted collaborative actions from the concerned stakeholders in Jordan.

## Introduction

Antimicrobial resistance (AMR) is a significant threat to global health. At least 700, 000 deaths occur yearly due to AMR (1). The antimicrobial stewardship (AMS) program is one of the key strategies that has been proposed by the World Health Organization (WHO) Global Action Plan to solve the problem of the inappropriate use of antimicrobials and hence tackle AMR (2). The WHO, through the Global Action Plan on AMR, put five strategic objectives for countries as a guiding principle in developing their own AMR national action plans: The objectives focused on improving awareness and understanding of AMR, strengthening evidence-based knowledge through surveillance and research, reducing the incidence of infection, optimizing the use of antimicrobial medicines, and increase investment in new drugs (3).

In response to this public health threat, Jordan formed, in 2017, a multi-sectoral high-level committee headed by the Ministry of Health (MOH) that launched a 4 years national AMR action plan (NAP) in Jordan (2018–2022) (4). Fulfilling the NAP objectives, Jordan took the first steps in 2018 towards establishing a national AMR surveillance system with the aim of estimating the burden and describing the trend of AMR to inform the national treatment guidelines of prevalent infectious diseases, enhance infection prevention and control (IPC) programs to curb the spread of AMR, as well as to design appropriate AMS programs (5).

Antimicrobial resistance is a serious threat to global health. Jordan, part of the Eastern Mediterranean Region (EMR), has a high prevalence of self-medication with antibiotics; recently reported as 40% (6). In response to the global call, in 2017, Jordan launched the NAP and the implementation commenced. The national AMR surveillance activities started in October 2018. Jordan’s AMR surveillance showed high resistance patterns of pathogens among inpatient and outpatient settings. High prevalence of extended spectrum beta-lactamase (ESBL) producers among *Escherichia coli* (47% in blood, 40% in urine) and *Klebsiella pneumonia* (51% in blood, 45% in urine) contributed to treatment failure using third-generation cephalosporins. This practice resulted in an increase in the number of prescriptions of carbapenems by physicians as a last resort in many complicated cases, which resulted in the emergence of carbapenem resistance which is more prominent in *Klebsiella pneumonia* (7–10).

Antimicrobial stewardship (AMS) is the optimization of the use of antimicrobials with the sole aim of improving patients’ health outcomes and reducing AMR while avoiding unnecessary treatment costs. Designing AMS programs is way far from waving a magic wand to create a ‘one-size fitsall’ program, yet it is a set of different complementary strategies with a common ultimate target; to curb the problem of antibiotic resistance (11). AMS has evolved over the years and is more developed in high-income countries (HICs) compared to low- and middle-income countries (LMICs). Several factors affect the implementation of AMS programs in hospitals. The size and types of care provided, as well as the complexity of antibiotic prescription, are all issues that are considered in designing an effective hospital-based program (12). Taking the special context of LMICs, the WHO has identified in 2019 six core elements to evaluate the AMS programs at the facility level in LMICs, including: (1) Leadership commitment, (2) accountability and responsibility, (3) AMS actions, (4) education and training, (5) monitoring and evaluation, and (6) reporting and feedback (13).

It is paramount to conduct a contextual evaluation of the efforts to implement AMS programs. There are numerous challenges to implementing a sustainable and effective AMS program, in Low-Middle Income Country (LMIC) contexts. It is important to understand these challenges so that the stewardship initiatives can be tailored according to the unique requirements thrown up by these healthcare facilities (14). Consequently, there is an imminent need to gather sufficient data to evaluate the core elements of AMS programs at Jordanian hospitals. Thus, its time to take a more structured approach and evaluate the AMS program core elements in Jordanian hospitals after the release of the national AMR action plan based on WHO directions. Therefore, this research aimed at assessing the implementation status of the AMS program in public hospitals in Jordan. It also aims at identifying the key barriers and challenges to the implementation of AMS interventions.

## Methods

### Study design and study population

A cross-sectional study by means of a face-to-face interview was conducted between the 1st of April 2022 and the 30^th^ of April 2022 with participants from the antimicrobial stewardship committees within the small, medium, and large public hospitals in Jordan. While public Hospitals with no established committee for antimicrobial stewardship were excluded. Therefore, 27 out of 32 public hospitals were approached. Private and Military hospitals were excluded due to the lengthy process to obtain approvals and the limited timeframe to conduct the research.

The primary data source was derived from the questionnaires filled in by the focal members of antimicrobial stewardship committees within the participating hospitals. The inclusion criteria comprised the small, medium, and large hospitals, as accredited and non-accredited hospitals within the public healthcare sector. Primary healthcare centers and ambulatory clinics were excluded.

The WHO Questionnaire was used as the research tool. It was distributed to 27 public hospitals that had an AMR committee, and a representative of the committee was interviewed and delegated to answer the questionnaire on behalf of the hospital.

### Sampling strategy

A total of 27 out of the 32 public hospitals were invited to participate in the study, while 5 were excluded due to the lack of an established AMS committee. Three investigators were assigned to contact the management in each hospital through the primary investigator. Each investigator was assigned a group of hospitals and interviewed a representative of the AMS committee in each hospital. In order to unify the interview approach and to exclude any bias, the three investigators met and discussed the questions and agreed upon the description of each question as per the guidance included within the WHO tool.

During the interview, the questionnaire was read and explained to each participant. The explanation was based on the detailed description included within the WHO tool. All participants voluntarily participated in the study and were thus considered exempt from written informed consent, which was written at the beginning of the tool before starting. The study’s aim and objectives were clearly explained at the beginning of the survey questionnaire.

### The questionnaire

The questionnaire was made up of three sections. The first section focused on participants’ background and demographic information (age, gender, educational level, and job position). The second section entailed questions that described and categorized the characteristics of the hospital (sector, geographic location, hospital size, accreditation status), and the scope of services (general, specialized). The third section dealt with the availability of AMS program core elements. In this section, a five-point Likert scale was employed, and 30 questions were further divided into six focus domains (Supplementary material). The domains are: (1) Leadership commitment, (2) accountability and responsibility, (3) AMS actions, (4) education and training, (5) monitoring and evaluation, and (6) reporting and feedback. The questionnaire was adopted from the WHO practical toolkit for antimicrobial stewardship programs in healthcare facilities in low- and middle-income countries (2019) (13). The tool asked the participants about the degree to which he/she agrees with the level of implementation and availability of each question within the six core elements. The participants’ responses ranged from 1 to 5, and the average score for each area was calculated, as well as the weighted average for each question.

### Ethical approval

All study participants gave their informed consent for inclusion before they participated in the study. The study protocol was approved by the Ministry of Health in Jordan (IRB approval no. 2232).

### Statistical analysis

Data was analyzed employing SPSS software, version 25. Descriptive statistics were used to describe the participants’ demographic characteristics. Data were reported as mean ± SD for normally distributed variables, while categorical data were reported as percentages (frequencies). Cronbach’s alpha was employed to measure the reliability of the tool with a set of 0.96 as the scale of reliability. Two-way ANOVA was employed to measure significance with a significance level set at 0.05.

## Results

### Background and demographics

A total of 27 healthcare providers representing the AMS committee participated in the study from 27 hospitals. Table 1 details the baseline characteristics of the participants. The majority of participants were females (85.2%) and bachelor’s degree holders (66.7%). When it comes to the experience of participants, 96% of them have more than 5 years of experience. All AMS committee focal points were either pharmacists (44.4%) or clinical pharmacists (55.6%).

**Table 1:** Demographic characteristics of participants.

Demographic parameter	Overall (*n* = 27) Frequency (%)
*Educational level*	
Bachelor’s degree	18 (66.7)
Postgraduate MSc	9 (33.3)
*Gender*	
Female	23 (85.2)
*Level of experience*	
1–5 years	1 (3.7)
6–10 years	9 (33.3)
11–15 years	13 (48.1)
More than 15 years	4 (14.8)
*Position*	
Pharmacist	12 (44.4)
Clinical Pharmacist	15 (55.6)

Table 2 presents the distribution of participants according to the demographics of their organizations. The majority of hospitals were in the middle region of Jordan (48.1%). Moreover, 19 (70.4%) hospitals were with be capacity ranging from 100 beds to 500 beds. In terms of scope of services, 21 (77.8%) were general hospitals. With regards to accreditation status, the hospitals were almost equally distributed among accredited (51.9%) and non-accredited (48.1%) hospitals.

**Table 2:** Participants’ hospitals characteristics (the distribution was based on participants responses).

Domain	Overall (*n* = 27) Number (%)
*Hospital size*	
Small (less than 100 beds)	7 (25.9)
Medium (100–500 beds)	19 (70.4)
Large (more than 500 beds)	1 (3.7)
*Region of the hospital*	
North	11 (40.7)
Middle	13 (48.1)
South	3 (11.1)
*Accreditation status*	
Accredited	14 (51.9)
Non-accredited	13 (48.1)
*Scope of service*	
General hospital	21 (77.8)
Specialized hospital	6 (22.2)

### Responses according to AMS program core elements

The assessment tool consisted of 30 questions based on the WHO’s six core domains of the AMS program. The results of AMS core elements application for all hospitals are showed in Table 3. The cut-off point was estimated by using the quartile percentile; P25 = **2.75**, P50 = **3.10**, and P75 = **3.60**. Therefore, the cut-off point needed to assume a good level of AMS application in the hospital was considered as P50 (**3.10**). Generally, most of the domains showed low application levels (below **3.10** out of **5**); the highest level was in AMS procedure application (actions; **3.61**), and the lowest application was in leadership commitment (**2.66**).

**Table 3:** The overall application average of antimicrobial stewardship (AMS) six core elements.

Domain	Application average (%)
Leadership commitment	2.66 (53)
Accountability and responsibilities	3.15 (63)
AMS actions	3.61 (72)
Education and training	2.85 (57)
Monitoring and surveillance	3.01 (60)
Reporting and feedback	2.96 (59)

Additionally, the correlation was used to explore the relationship between the availability of AMS core elements and other hospital characteristics such as capacity (large, medium, small), location (north, center, and south), the scope of work (general or specialized), or if the hospital was accredited or not. Generally, the analysis showed that there were no statistical correlations between the availability of core elements for the AMS program and these variables and there was no significant difference among those variables.

Table 4 details the level of application of each question under each core domain. Within the first core domain **(leadership Commitment)**, the highest application within hospitals was for having an AMS action plan (65.86%). While prioritizing the AMS program scored (59.2%) and dedicating financial support scored the lowest (34.8%). For the second core element **(Accountability and Responsibility)**, results showed a score of 74% among participating hospitals for the availability of a multidisciplinary AMS leadership committee with clear terms of reference. However, the presence of clearly defined collaboration between the AMS and IPC programs scored low (51.8%). The availability of **AMS actions** was the highest-scoring core element among the six AMS areas (72%). Results showed that almost all participating hospitals have a formulary with a list of approved antibiotics. When it comes to **Education and Training,** the overall compliance was low (57%) among all participating hospitals. Provision of basic training in optimal antibiotic use for healthcare professionals scored only (48.84%). Looking at the availability of the **Monitoring and Evaluation** core element, results showed that the monitoring of antibiotic susceptibility and resistance rates was scored below average (59.2%). As for the monitoring of the quantity and types of antibiotic use at the unit and/or facility-wide level, it was poorly applied scoring 67.34%. The analysis of the **Reporting and Feedback** element showed that regular evaluation and sharing of resistance rates were poorly conducted with a score of 56.98%. Developing an antibiogram for key antibiotics attained a low score that did not exceed 59.2%.

**Table 4:** Participants’ responses regarding the AMS core elements.

No.	Domain	Strongly disagree frequency (%)	Disagree frequency (%)	Do not know frequency (%)	Agree frequency (%)	Strongly agree frequency (%)	Total score (%)
	Leadership commitment						
1.1.	AMS program is identified as a priority for health-care facility management	4 (14.8)	5 (18.5)	7 (25.9)	10 (37.0)	1 (3.7)	59.2
1.2.	Health-care facility has AMS action plan that prioritizes activities and measures progress and accountability	5 (18.5)	3 (11.1)	2 (7.4)	13 (48.1)	4 (14.8)	65.86
1.3.	There are dedicated financial support for the health-care facility AMS action plan	16 (59.3)	4 (14.8)	5 (18.5)	2 (7.4)	0 (0)	34.8
2.	Accountability and responsibility						
2.1.	A multidisciplinary AMS leadership committee in place with clear terms of reference	3 (11.1)	3 (11.1)	3 (11.1)	8 (29.6)	10 (37.0)	74
2.2.	A dedicated AMS leader/champion is identified within the health-care facility	5 (18.5)	2 (7.4)	6 (22.2)	8 (29.6)	6 (22.2)	65.86
2.3.	The multidisciplinary AMS team is in place with terms of reference	4 (14.8)	3 (11.1)	9 (33.3)	6 (22.2)	5 (18.5)	63.64
2.4.	Other health professionals are identified and involved in AMS activities	5 (18.5)	4 (14.8)	3 (11.1)	14 (51.9)	1 (3.7)	61.96
2.5.	There is a clearly defined collaboration between the AMS and IPC programs	6 (22.2)	7 (25.9)	7 (25.9)	6 (22.2)	1 (3.7)	51.80
2.6.	There are regular (descriptive) activity reports on the implementation of the AMS program	4 (14.8)	7 (25.9)	5 (18.5)	5 (18.5)	6 (22.2)	61.42
3.	AMS actions						
3.1	There are up-to-date standard treatment guidelines	4 (14.8)	1 (3.7)	5 (18.5)	13 (48.1)	4 (14.8)	68.82
3.2.	Regular AMS team review/audit of specified antibiotic therapy or clinical conditions is conducted at the healthcare facility	2 (7.4)	1 (3.7)	9 (33.3)	13 (48.1)	2 (7.4)	68.82
3.3.	Advice/feedback from AMS team members is easily accessible/available to all prescribers	3 (11.1)	1 (3.7)	2 (7.4)	17 (63)	4 (14.8)	73.34
3.4.	The AMS team conducts regular ward rounds and other AMS interventions in select health-care facility departments	3 (11.1)	2 (7.4)	6 (22.2)	16 (59.3)	0 (0)	65.94
3.5.	Health-care facility has a formulary with a list of approved antibiotics	1 (3.7)	1 (3.7)	1 (3.7)	11 (40.7)	13 (48.1)	85.1
3.6.	Health-care facility has a list of restricted antibiotics	2 (7.4)	3 (11.1)	2 (7.4)	10 (37)	10 (37)	76.96
3.7.	Laboratory and imaging services are accessible to support AMS interventions	6 (22.2)	2 (7.4)	7 (25.9)	8 (29.6)	4 (14.8)	61.42
3.8.	Health-care facility has access to IT services to support AMS activities	5 (18.5)	4 (14.8)	7 (25.9)	9 (33.3)	2 (7.4)	59.2
3.9.	There is a standardized facility prescription chart and medical records	0 (0)	2 (7.4)	8 (29.6)	17 (63.0)	0 (0)	71.12
3.10.	There is a Health-care facility policy for documenting prescribed medicines	1 (3.7)	4 (14.8)	1 (3.7)	17 (63.0)	4 (14.8)	74.08
4	Education and training						
4.1.	Basic training in optimal antibiotic use is provided for health-care professionals	7 (25.9)	9 (33.3)	5 (18.5)	4 (14.8)	2 (7.4)	48.84
4.2.	Continued training in optimal antibiotic use is provided for health-care professionals	7 (25.9)	9 (33.3)	5 (18.5)	5 (18.5)	1 (3.7)	48.1
4.3.	Initial and regular training of the AMS team in infection management is provided	5 (18.5)	9 (33.3)	8 (29.6)	5 (18.5)	0 (0)	49.58
5	Monitoring and evaluation						
5.1.	There is monitoring of the appropriateness of antibiotic use at the unit and/or facility-wide level through audits or PPSsi	2 (7.4)	7 (25.9)	8 (29.6)	7 (25.9)	3 (11.1)	61.42
5.2.	There is monitoring of the quantity and types of antibiotic use (purchased/prescribed/dispensed) at the unit and/or facility-wide level	2 (7.4)	4 (14.8)	8 (29.6)	8 (29.6)	5 (18.5)	67.34
5.3.	There is monitoring of antibiotic susceptibility and resistance rates for a range of key indicator bacteria	6 (22.2)	4 (14.8)	4 (14.8)	11 (40.7)	2 (7.4)	59.2
5.4.	There is monitoring of the compliance with AMS interventions by the AMS committee	4 (14.8)	5 (18.5)	7 (25.9)	10 (37.0)	1 (3.7)	59.2
6	Reporting and feedback						
6.1.	Regular evaluation and sharing of health-care facility data on antibiotic use with prescribers is done	4 (14.8)	4 (14.8)	6 (22.2)	10 (37.0)	3 (11.1)	63.54
6.2.	Regular evaluation and sharing of health-care facility resistance rates with prescribers is done	5 (18.5)	3 (11.1)	13 (48.1)	3 (11.1)	3 (11.1)	56.98
6.3.	Evaluation of appropriateness of data on antibiotic use is shared with prescribers	4 (14.8)	5 (18.5)	8 (29.6)	5 (18.5)	5 (18.5)	61.42
6.4.	Health-care facility develops antibiogram for key antibiotics informed by data on antibiotic use and resistance	5 (18.5)	5 (18.5)	5 (18.5)	10 (37.0)	2 (7.4)	59.2

## Discussion

This study explored the current status of the implementation of the AMS program in public hospitals in Jordan. Overall, 84.4% of the public-hospital (27 out of 32) established an AMS committee, which was the target of this study. Using the WHO’s core elements, we found that compliance ranged from 52 to 72%, and hospitals reported having an AMS program that implemented all the core elements defined by the WHO in 2018 in response to the national action plan (4). Four years after starting the implementation of the NAP, the results of the national AMR surveillance program reflect alarmingly rising levels of multidrug-resistant pathogens (15). The findings of the surveillance reports clearly revealed the scale of the problem, yet the effectiveness of solutions and interventions that have been put in place 4 years ago is not clearly demonstrated.

In 2017, the implementation of the AMS program core elements, as aligned by the CDC, was evaluated in Jordanian Hospitals. Ababneh et al. conducted a cross-sectional study in 41 Jordanian hospitals regarding adherence to the CDC criteria for the AMS program. Among the enrolled hospitals in the study, 17.1% had an infectious diseases specialist as the head of AMS, whereas 73.2% had clinical pharmacists involved as AMS leaders (16). In comparison, the results of the current study revealed interesting findings pertinent to the structure of the AMS committee. All public hospitals had either a pharmacist or clinical pharmacist leading the AMS program. Despite variations among countries in terms of AMS committee structure, the consensus is that pharmacists/ clinical pharmacists are a fundamental part of the committee (17–19). Their role can entail developing and managing guidelines, education, monitoring compliance with antimicrobial use and auditing outcome of use (18, 20, 21). However, the role of pharmacists in Jordan was explored due to the shortage of medical professional expertise. Several studies reported that pharmacists expressed difficulties conveying their opinions and recommendations on antibiotic therapy to physicians despite frequent communications (22). Thereover, it is vital to empower pharmacists to lead AMS interventions and drive change.

The findings in this study showed an alarming result when it relates to leadership commitment, particularly in terms of the provision of financial support with a total score of 34.8%. In fact, AMS programs in hospitals need significant funding support, trained human resource, and political will (23–25). A robust level of implementation of stewardship measures in a hospital requires a committed team of experts; and the support of microbiology laboratories and hospital information systems (26). In the context of Jordanian hospitals, very little effort has been taken to look at the feasibility of implementing AMS interventions (27). The interventions that are feasible and effective in low-resource settings, may be different from those which has succeeded in larger hospitals situated in high-income countries (14). Similar findings were reported as a key challenge to effective AMS programs (28, 29) even in high-income countries like the United States (30). A study in Indonesia reported that less than 50% of hospitals allocated funds to support AMS programs (28). A multinational group of experts among EU and US hospitals agreed on core indicators to assess the AMS programs, financial support to the provision of salary funding for dedicated support of AMS activities was considered a core indicator (30). Similar results were also reported in the study of Ababneh et al. (16) where none of the assessed facilities presented a financial report endorsing ASP responsibilities.

Two encouraging findings of this study are associated with the two key questions in the action domain, which received the highest mean score: the availability of a formulary with a list of approved antibiotics (85.1%) and the availability of an approved list of restricted antibiotics (76.96%). However, the lack of adequate audit (68.82%) prevents the feasible evaluation of the effectiveness of these processes. Unfortunately, the antibiotic restriction may not stop the possible overuse of existing broad-spectrum antimicrobials (31). The availability of structure indicators is vital in AMS programs, however, process and outcome indicators of the program were not encouraging (16, 32). Particularly if that was not accompanied by continuous education and training (32, 33).

In this study, education and training scored below average (57%). Literature also supports that investment in education and training can substantially improve the outcome (23–25, 30, 34). The next logical step is to translate commitment and education into actions. Several studies reported that the main source of the misuse of antimicrobials is a lack of knowledge among healthcare professionals (11, 13, 34). A cross-sectional study in Jordan in 2021, focused on identifying the perceptions and practices among Jordanian healthcare practitioners toward AMS programs. The findings revealed a positive perception towards the program, while practices pertinent to this element were suboptimal. Further, the results showed that longer years of practice, postgraduate studies, and practice in academia sectors yielded higher perception scores (*p* value = 0.0335, 0.0328, and 0.0007 respectively) (35). Therefore, it is now vital that both academia and the MOH cooperatively focus on integrating antimicrobial resistance and good practices in all healthcare professional curricula and in-service educational sessions (34). Such educational sessions need also to include community awareness. A study by Alzoubi and coworkers (36), reported a low average knowledge about the use of antibiotics among 1,091 Jordanian patients attending outpatient clinics. Only 20.1% of the participants stated that antibiotics were used for bacterial infections. Moreover, several studies revealed that the prevalence of self-medication with antibiotics in Jordan remains high (37, 38), this in turn counteracts efforts of AMS in hospitals and leads to them being ineffective. Enforcement of legislation may need to be pursued to enable successful AMS programs in Jordan.

Another prominent challenge that was identified in this study was the lack of collaboration. This was evident from the answers to the following question “*There is a clearly defined collaboration between the AMS and IPC programs*” (score 51.8%). Effective AMS programs required a multifaceted collaboration within the organization and even outside it (39). Collaboration between IPC and AMS committees can enhance performance between the two parties, produce synergistic actions and mitigate any impediments (25, 40). Collaboration between the two committees has it rational as both serve a common purpose and they share similar expertise. The earlier study in Jordan (16) reported that the personnel who were most collaborative with the AMS team were clinicians (51.2%) and the least collaborative were microbiologists (17.1%). Therefore, this lack of collaboration will impact the effectiveness of the AMS program.

Access to IT services, laboratory, and imaging services to support AMS activities were scored low, 61.42, and 59.20%, respectively. Delays in service provision are critical for effective clinical outcome. Several reports identified the importance of timely intervention and that the reduction in the use of antibiotics is not the key attribute, reduction of inappropriate use and timely access to effective treatment is the key indicator for effective AMS programs (24, 41). Limited access imparts timeliness to the provision of care. Effective AMS program requires regular updates on relevant information that may include resistant bacteria, the incidence of antibiotic-resistant bacteria which requires easy access to IT and laboratory services (42). The IT services need to be integrated within the AMS program due to their role in the provision of support for decisions concerning antibiotic prescriptions, offering facilities for the collection and reporting of antibiotic use, as well as providing information and protocols that can be directly linked to AMS program or clinical guidelines, hence, possibly improving the rational use of antimicrobials (28, 43). An example of the use of technology that supported AMS programs is the implementation of automatic stop order (ASO), which is considered one of the technological tools by which identified medications are re-evaluated and reviewed on a consistent basis to ensure preventing unreasonable and prolonged use of drugs. With ASO, stop dates are automatically applied to an antimicrobial order when the duration of therapy is not specified. The goal is to ensure that antimicrobials are continued no longer than necessary. ASO encourages reassessment of the duration of therapy based on the patient’s response to treatment, and prescriber review of laboratory, microbiology and diagnostic imaging results after the specified length of time (44). In Jordan, the use of ASO is in its infancy.

Monitoring and evaluation (overall score of 60%), as well as reporting and feedback (overall score 59%) were also scored low in this study. Jordan public hospitals are still in the early stages of implementation and a lot of efforts need to be made to comply with such aspects. In comparison with other countries, A study in acute care hospitals in the United States reported that 79.3% of surveyed hospitals complied with monitoring of prescribing and antibiotic resistance patterns (30). Several countries employed pharmacists and clinical pharmacists to lead the monitoring and evaluation processes of the AMS programs (17–19). In a study in India, pharmacist-led model over 1 year resulted in an increase in prescribed antibiotic appropriateness from 56 to 80% and compliance to recommendations increased from 54 to 70% (21). In reference to that, as pharmacists/clinical pharmacists are leading the AMS program in Jordan, it is proposed that they are empowered to lead the monitoring and evaluation processes.

### Implication of the study

The WHO core elements for the assessment of the effectiveness of AMS programs needs to be employed to conduct periodic cross-sectional audits for the public and private sector in Jordan. This will enable tracking the progress of the implementation of the program across the country and identifying gaps within the practice. Jordan has made commitments to curb antimicrobial resistance using the NAP (4). Nevertheless, the findings of this study raise apprehensions over the implementation gaps in rendering the political commitment of the MOH into favorable actions. Consequently, the findings of this study set as a reminder to accelerate the implementation of NAP that entails AMS programs in various healthcare settings in Jordan and other LMICs. There is a need to expand training and professional education on AMR, MoH could consider focusing efforts on physicians. A recent study found that one-third of physicians reported no knowledge of any initiatives on antibiotic awareness and resistance and nearly 90% were unaware or unsure of the existence of a NAP on AMR (45). Additionally, IPC compliance and effectiveness needs to be regularly assessed in health facilities. Further, sufficient and consistent financing is key to the sustainable implementation of AMR actions in Jordan, therefore budget allocation for the implementation of AMS program is needed within each hospital.

Additionally, it is important to further explore barriers and facilitators of implementing interventions to improve antimicrobial stewardship in Jordan through a qualitative study to investigate the real implementation status of AMS program. A study reported that in 2019, approximately 59% of the antibiotics consumed nationally were from the WHO’s AWaRe classification of antibiotics (46, 47). Therefore, future study needs to consider evaluating the class of antibiotic usage.

### Strengths and limitations

To the best of our knowledge, this is the first study in Jordan that assess the AMS program after 4 years of the implementation of the NAP in 2017 using the WHO core elements for the AMS program for LMIC. There is a limited number of publications in LMIC assessing the effectiveness of the AMS program. However, this study had a few limitations. First, the use of self-reported data is subject to bias. Some of the participants may have overestimated or underestimated their responses to the questionnaire, impacting the accuracy of the findings. Second, the military and private hospitals were not included and therefore, the generalizability of the results is not feasible. Third, no pre-implementation data were available to attribute the observed results to the AMS program. Despite that, in terms of impact, the study described specific gaps in the AMS program in public hospitals in Jordan. These results are similarly useful for other hospitals in Jordan (private or military) as well as other LMICs. An additional limitation is pertinent to the limited number of studies that evaluated AMS programs in LMICs using the WHO core elements, particularly in the middle east, which limited our ability to compare our findings with similar programs. Owing to the study design (questionnaire with closed-end answers), we might have missed some information that could be captured using open end questions.

## Conclusion

The current study elaborated on the level of implementation of the AMS program in public hospitals in Jordan, using the WHO core elements for the evaluation of the AMS program at the facility level in LMICs. Overall, the level of implementation of the program was not optimal and there is still much more that needs to be done. Key gaps were identified pertinent to the provision of financial support, lack of training, poor collaboration, and inadequate access to IT and diagnostic services. A well-structured monitoring, and evaluation processes as well as feedback provision were below the average. Leadership and key stakeholders’ commitment and support underpin the success of the AMS program. Overall, the results provided a baseline to monitor progress toward the national AMR action plan (NAP) in Jordan.

## Data availability statement

The original contributions presented in the study are included in the article/Supplementary material, further inquiries can be directed to the corresponding author.

## Ethics statement

All study participants gave their informed consent for inclusion before they participated in the study. The study protocol was approved by the Ministry of Health in Jordan (IRB approval no. 2232).

## Author contributions

SH: conceived the study and project management. ED: led writing, reviewing and editing the manuscript, and validation of results. TM: conceptualization and project supervision. OT: implementation of the research and statistical analysis. TJ and WA: data collection and implementation of the research. RG: study design and implementation of the research. ZH: implementation of the research. All authors contributed to the article and approved the submitted version.

## Funding

The study was supported by the Healthcare Accreditation Council, Amman, Jordan.

## Conflict of interest

The authors declare that the research was conducted in the absence of any commercial or financial relationships that could be construed as a potential conflict of interest.

## Publisher’s note

All claims expressed in this article are solely those of the authors and do not necessarily represent those of their affiliated organizations, or those of the publisher, the editors and the reviewers. Any product that may be evaluated in this article, or claim that may be made by its manufacturer, is not guaranteed or endorsed by the publisher.
