# Supplementary material for: Four years after the implementation of antimicrobial stewardship program in Jordan: evaluation of program’s core elements
Source: Front Public Health. 2023 May 30;11:1078596. doi: 10.3389/fpubh.2023.1078596 (PMC10262748; doi:10.3389/fpubh.2023.1078596)
Supplement: Supplementary file 1 [file Table_1.DOCX]

| **#** | **Requirements** | **Description** | **Rating** | | | | |
| --- | --- | --- | --- | --- | --- | --- | --- |
|  |  |  | **Strongly Disagree** | **Disagree** | **Don’t Know** | **Agree** | **Strongly Agree** |
| **1** | Antimicrobial Stewardship is identified as a priority for health-care facility management | The facility management has formally identified AMS as a priority objective for the facility and included it in its key performance indicators. Financial and human resources have been allocated for AMS activities. |  |  |  |  |  |
| **2** | Health-care facility has an action plan for Antimicrobial Stewardship, which prioritizes activities and measures progress and accountability | A health-care facility AMS action plan is endorsed that prioritizes activities and measures progress and accountability for ensuring appropriate antibiotic use, based on existing national or international guidelines and/or an existing national strategy. The AMS action plan is updated regularly as required. |  |  |  |  |  |
| **3** | Financial support is dedicated for the health-care facility AMS action plan | There is dedicated, sustainable and budgeted financial support for AMS activities in the action plan (e.g. support for salary, training and information technology (IT) support). |  |  |  |  |  |
| **4** | A Multidisciplinary AMS leadership committee is in place with clear terms of reference | This AMS committee can be either stand-alone or embedded in another existing committee structure (e.g. drug and therapeutics committee, pharmacy committee, infection control committee, patient safety committee). If embedded in another committee, AMS must be a standing item on the committee’s agenda. The AMS committee is explicitly in charge of setting and coordinating the AMS programme/strategy according to its terms of reference. |  |  |  |  |  |
| **5** | A Dedicated AMS leader/champion is identified for the health-care facility | A health-care professional has been identified as a leader/champion for AMS activities at the facility and is responsible for leading the AMS team in implementing the AMS programme. |  |  |  |  |  |
| **6** | There is a multidisciplinary AMS team with terms of reference | An AMS team of multidisciplinary health-care professionals who will implement the day-to-day AMS activities in the health-care facility. In resource-limited settings or small facilities it is often difficult to have an AMS team, and an AMS champion can be identified instead. The composition of the AMS team is flexible and should be based on existing recommendations and adapted to the local context: • option 1: >2 health-care professionals constituting a multidisciplinary team (e.g. tertiary hospitals); • option 2: a prescriber and a nurse or pharmacist (e.g. secondary or small hospitals); or • option 3: an AMS champion, e.g. a physician, nurse or pharmacist leading the stewardship programme, with access to expert advice |  |  |  |  |  |
| **7** | Other health professionals are identified and involved in AMS activities | Other health-care professionals apart from the AMS team (e.g. from the ICU, internal medicine and surgery, health informatics, or pharmacy or nursing personnel) participate in AMS activities based on the priorities of the health-care facility AMS action plan |  |  |  |  |  |
| **8** | There is a clearly defined collaboration between the AMS and IPC programs | A document clearly specifies the process of collaboration between the AMS team/committee and the IPC programme and/or committee. In many low-resource settings the IPC and AMS committees may be merged into one. |  |  |  |  |  |
| **9** | Regular activity reports are produced and disseminated on the implementation of the AMS program | Regular activity reports are produced and disseminated to health-care facility personnel and regional/ national AMS TWGs. These reports include data on antibiotic use/consumption and describe the interventions implemented by the AMS team. |  |  |  |  |  |
| **10** | The health-care facility has available, up-to-date recommendations for infection management based on international/national evidence-based guidelines and local/national susceptibility patterns (where possible) | The health-care facility has available, up-to-date recommendations for infection management based on international/national evidence-based guidelines and local/national susceptibility patterns (where possible), to assist with antibiotic selection for common clinical conditions (indication, agent, dose, route, interval, duration). A process is in place for regular review and updating of the guidelines based on new evidence or other external input |  |  |  |  |  |
| **11** | Regular AMS team review/audit of specified antibiotic therapy or clinical conditions at the healthcare facility | Depending on available resources, this can be conducted by prioritizing wards or specific patient conditions |  |  |  |  |  |
| **12** | Advice/feedback from AMS team members is easily accessible/available to all prescribers | This can be achieved through various methods, including facility ward rounds, bedside consultations and dedicated telephone lines. |  |  |  |  |  |
| **13** | The AMS team conducts regular ward rounds and other AMS interventions in select health-care facility departments | The AMS team conducts regular ward rounds (in one or more wards) and other AMS interventions in select facility departments (one or more) identified in the health-care facility AMS action plan. |  |  |  |  |  |
| **14.1** | The health-care facility has a formulary with a list of approved antibiotics that may be based on national recommendations or the WHO EML | The health-care facility has a formulary with a list of approved antibiotics that may be based on national recommendations or the WHO EML |  |  |  |  |  |
| **14.2** | Health-care facility formulary with a list of restricted antibiotics. | The health-care facility has a formulary with a list of antibiotics approved for use in the facility and specifies a list of restricted antibiotics that require approval by the designated AMS team member (or infectious disease physician if available, physician or AMS champion) when used and/or are only permitted for specific conditions, e.g. the WATCH and RESERVE groups of antibiotics. |  |  |  |  |  |
| **15** | The health-care facility has access to (on-site or off-site) laboratory and imaging services to support AMS interventions | The health-care facility has access to (on-site or off-site) laboratory and imaging services, and to timely, quality-assured results to support diagnosis of the most common infections. |  |  |  |  |  |
| **16** | Health-care facility has access to IT services to support AMS activities. This could include, for example, measurement of antibiotic use. | The specific requirements need to be defined at local/regional/national level. This could include, for example, measurement of antibiotic use. |  |  |  |  |  |
| **17** | The health-care facility ensures the availability and use of standardized prescription charts, medical records and transfer notes | Standardized facility prescription chart and medical records |  |  |  |  |  |
| **18** | The health-care facility has a written policy that requires prescribers to clearly document the indication and antibiotics prescribed (agent, dose, route, interval, duration and review dates) in the prescription chart, medical record and transfer notes to other health-care institutions | The health-care facility has a written policy that requires prescribers to clearly document the indication and antibiotics prescribed (agent, dose, route, interval, duration and review dates) in the prescription chart, medical record and transfer notes to other health-care institutions. |  |  |  |  |  |
| **19.1** | The health-care facility offers Basic training in optimal antibiotic use for health-care professionals | The health-care facility offers basic induction training (e.g. sensitization on AMR and use of standard treatment guidelines) to staff on how to optimize antibiotic prescribing, dispensing and administration. |  |  |  |  |  |
| **19.2** | The health-care facility offers continued educational resources to train health-care professionals on the optimal antibiotic use | The health-care facility offers continued educational resources (e.g. regular training on infection management) to train staff on how to optimize antibiotic prescribing, dispensing and administration. |  |  |  |  |  |
| **20** | The health-care facility offers initial and regular training of the AMS team in infection management (diagnosis, prevention and treatment) | The health-care facility offers initial and regular training of the AMS team in infection management (diagnosis, prevention and treatment) and AMS. This training is usually not offered at the facility level, but is likely to be available at the regional, national or international level. The facility should, however, ensure that members of the AMS team are adequately trained, according to local/national requirements. |  |  |  |  |  |
| **21** | The AMS team undertakes audits to assess the appropriateness of infection management and antibiotic prescription | The AMS team undertakes audits or PPSs, at the unit and/or health-care facility level, to assess the appropriateness of infection management and antibiotic prescription (e.g. indication, agent, dose and duration of antibiotic therapy in specific infectious conditions such as pneumonia or surgical prophylaxis) according to policy/guidance. |  |  |  |  |  |
| **22** | Monitoring quantity and types of antibiotic use (purchased/prescribed/dispensed) at the unit and/or facility-wide level | In collaboration with the facility pharmacy, the AMS team monitors the quantity and types of antibiotic use (purchased/prescribed/dispensed) at the unit and/or health-care-facility level |  |  |  |  |  |
| **23** | Monitoring of antibiotic susceptibility and resistance rates for a range of key indicator bacteria | The AMS team monitors antibiotic susceptibility and resistance rates for a range of key indicator bacteria at the health-care facility-wide level, in alignment with national and/or international surveillance systems (e.g., GLASS). |  |  |  |  |  |
| **24** | Monitoring compliance of AMS interventions by the AMS committee | The AMS committee monitors compliance with one or more of the specific interventions put in place by the AMS team (e.g., indication captured in the medical record for all patients on antibiotics) |  |  |  |  |  |
| **25** | Regular evaluation and sharing of health-care facility data on antibiotic use with prescribers | Health-care-facility reports on the quantity of antibiotics purchased/prescribed/dispensed are reviewed and analyzed, and key findings are shared with prescribers along with specific action points. |  |  |  |  |  |
| **26** | Regular evaluation and sharing of health-care facility resistance rates with prescribers | The facility reports on antibiotic susceptibility rates are reviewed, and analyses and key findings are shared with prescribers along with specific action points. |  |  |  |  |  |
| **27** | Evaluation of appropriateness of data on antibiotic use is shared with prescribers | Findings from audits/reviews of the quality/appropriateness of antibiotic use are communicated directly to prescribers along with specific action points. |  |  |  |  |  |
| **28** | Health-care facility antibiogram for key antibiotics informed by data on antibiotic use and resistance | The health-care facility aggregate antibiogram is developed and regularly updated based on a review and analysis of facility antibiotic use and antibiotic-resistant bacteria. The antibiogram may help to inform updates of clinical guidelines. |  |  |  |  |  |
